# Transdermal Delivery of Glimepiride: A Novel Approach Using Nanomicelle-Embedded Microneedles

**DOI:** 10.3390/pharmaceutics15082019

**Published:** 2023-07-26

**Authors:** Sadia Pervez, Fazli Nasir, Talaya Hidayatullah, Muzna Ali Khattak, Fawaz Alasmari, Syeda Rabqa Zainab, Shazma Gohar, Arbab Tahir, Gul e Maryam

**Affiliations:** 1 Department of Pharmacy, University of Peshawar, Peshawar 25000, Pakistan; sadiapervez@uop.edu.pk (S.P.); talayaarbab@uop.edu.pk (T.H.); muznaali@uop.edu.pk (M.A.K.); syedarabqazainab@uop.edu.pk (S.R.Z.); shazmagohar@gmail.com (S.G.); tahir.arbab@uop.edu.pk (A.T.); 2 Department of Pharmacology and Toxicology, College of Pharmacy, King Saud University, Riyadh 11451, Saudi Arabia; ffalasmari@ksu.edu.sa; 3 Department of Pharmacy, Qurtaba University of Science and Information Technology, Peshawar 25000, Pakistan; gulemaryam112@gmail.com

**Keywords:** glimepiride, BCS class II drug, transdermal drug delivery, microneedles, nanomicelles, Soluplus^®^, particle size distribution, pharmacokinetic parameters

## Abstract

Glimepiride (GM) is a hydrophobic drug that dissolves slowly and yields inconsistent clinical responses after oral administration. Transdermal drug delivery (TDD) is an appropriate alternative to oral administration. Microneedles (MNs) offer a promising delivery system that penetrates the skin, while polymeric micelles can enhance the solubility; hence, the combination of both results in high drug bioavailability. This study aims to improve glimepiride’s solubility, dissolution rate, and bioavailability by incorporating nanomicelles into MNs for TDD. The nanomicelles formulated with 10% Soluplus^®^ (SP) and 40% GM had a mean particle size of 82.6 ± 0.54, PDI of 0.1 ± 0.01, −16.2 ± 0.18 zeta potential, and achieved a 250-fold increase in solubility. The fabricated pyramid shaped GM-dissolving MNs were thermally stable and had no formulation incompatibility, as confirmed by thermal and FTIR analysis. The in vitro dissolution profile revealed that the GM release from nanomicelles and nanomicelle-loaded DMN was concentration-independent following non-Fickian transport mechanism. Improved pharmacokinetic parameters were obtained with dose of 240 µg as compared to 1 mg of GM oral tablet, in healthy human volunteers. The observed C_max_, T_max_ and MRT were 1.56 μg/mL ± 0.06, 4 h, and 40.04 h ± 3.37, respectively. The safety profile assessment indicated that microneedles are safe with no adverse effects on skin or health. This study provides an alternative delivery system for the administration of glimepiride, resulting in improved bioavailability, enhanced patient compliance, and reduced dosing frequency.

## 1. Introduction

Diabetes mellitus (DM), a chronic endocrine disorder, caused by insufficient production or abnormal secretion of insulin from the pancreas. This metabolic condition is characterized by elevated blood glucose levels also known as hyperglycemia [1]. Diabetes is of two types: Type I DM, also called insulin-dependent diabetes, or childhood-onset diabetes and Type II DM, also called adulthood diabetes or non-insulin-dependent diabetes, which is linked to obesity, insulin resistance, and defective beta cell function [2]. Type II DM accounts for approx. 90% of all diabetic cases [3]. Drug therapy is recommended in cases where lifestyle modifications and counseling fail to achieve desired therapeutic outcomes in noninsulin-dependent DM. Oral anti-diabetic drugs such as sulfonylureas are the most commonly prescribed drugs in such cases [4].

Glimepiride (GM) is a third-generation anti-diabetic drug belonging to the sulfonylurea class. It is used to treat type II DM by acting as an insulin secretagogue to lower blood sugar levels.

**Figure 1:**
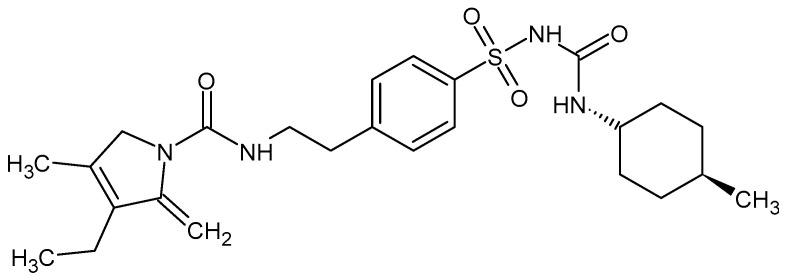
Glimepiride chemical structure.

Glimepiride as shown in Figure 1 has the chemical formula C_24_H_34_N_4_O_5_S and a molecular weight of 490.6 g/mol. Its International Union of Pure and Applied Chemistry (IUPAC) name is *N*-[4-[2-(3-ethyl-4-methyl-2-oxo-3-pyrroline-1-carboxamido)-ethyl]-benzenesulfonyl]-*N′*-4-methylcyclohexylurea [5]. GM is a crystalline, odorless, and white to yellowish white substance that is almost insoluble in water.

However, GM’s oral administration has some drawbacks, including the risk of hypoglycemia in the first few hours and irregular bioavailability due to limited water solubility, leading to poor patient adherence [6]. GM, a BCS class II drug, owing to its low solubility that may result in suboptimal plasma drug levels, inconsistent therapeutic outcomes, and high inter-individual variability [7]. Nevertheless, various strategies such as solid dispersion methods, water-soluble polymers, and micelles are used to enhance the drug’s solubility and improve its clinical effectiveness [8]. These methods have issues such as compatibility, uneven distribution, limited drug-loading capacity, potential leakage, alterations in drug release profiles, and dependency on micellar stability [9,10,11].

The limitations inherent in traditional drug delivery systems have highlighted the necessity for alternative routes. The limitations include limited drug solubility, poor bioavailability, inadequate drug targeting to specific sites, lack of sustained or controlled release, systemic side effects, and low patient compliance due to frequent dosing requirements [12]. As a result, efforts have been directed towards improving transdermal drug delivery systems as a potential solution to the shortcomings associated with conventional methods of administering antidiabetic drugs [12].

Transdermal drug delivery (TDD) presents an attractive alternative to conventional techniques due to its ease of accessibility, painlessness, self-administration convenience, and ability to bypass presystemic metabolism, which enhances bioavailability [12]. However, one obstacle in developing TDD systems is overcoming the natural transport barrier of the skin, which is the stratum corneum (SC) [13]. To enhance drug absorption, various active and passive technologies are employed, including passive techniques such as use of chemical enhancers and emulsions and active methodologies like ultrasound, velocity-based devices (powder or jet injectors), electrically operated methods (iontophoresis and electroporation), thermal assistance (such as radio-frequency heating and laser), and mechanical methodologies such as tape stripping and microneedles (MN) [12].

Microneedles have emerged as a popular research topic lately because of their potential to offer an enhanced approach to drug delivery as compared to current TDD methods [14]. These microneedles are designed with adequate length for effective penetration of the outer layer of the skin (stratum corneum) while being precisely sized to minimize any discomfort or pain. Dissolving microneedles are a promising drug delivery technology that can deliver drugs painlessly and effectively through the skin [15]. Dissolving microneedles (DMNs) are tiny needles containing drugs that release medication in the skin for local or systemic delivery over varying durations. Unlike biodegradable microneedles, DMNs can hold higher drug doses but require low drug solubility or encapsulation in a controlled release polymer for sustained drug release in the skin layers after dissolution, which occurs within minutes. Glimepiride’s low aqueous solubility makes it a potential candidate for sustained dissolution in the skin via DMNs [16].

Nanomicelles are self-assembled nanoscale structures formed by amphiphilic molecules, offering valuable applications in drug delivery. They can encapsulate hydrophobic drugs, improving solubility for targeted delivery [17]. Alongside liposomes, dendrimers, and nanoparticles, nanomicelles have revolutionized drug delivery by enabling sustained, controlled, and targeted release of therapeutic agents. Their easy preparation, biocompatibility, efficacy, and ability to encapsulate poorly water-soluble drugs have gained significant attention [18]. Soluplus^®^ is a common excipient used in transdermal drug delivery to enhance skin penetration. It acts as a permeation enhancer by reducing the barrier function of the skin, modifying the lipid bilayer fluidity, and forming micelles to encapsulate and increase drug solubility [19,20]. These mechanisms facilitate drug diffusion, making Soluplus^®^ a useful excipient for improving transdermal drug delivery [21].

Soluplus^®^, a water-soluble polymer, is also used as a surfactant, solubilizer, and emulsifier in microneedle fabrication. It can also act as a binder for stable microneedle production while enhancing drug solubility and permeability. Soluplus^®^ is well-suited for microneedle drug delivery due to its biocompatibility, non-toxicity, and skin tolerance [22]. Polyvinyl alcohol (PVA) and polyvinylpyrrolidone (PVP) are water-soluble and biocompatible polymers used as matrix-forming materials in microneedle patch production [23]. They contribute as binders, plasticizers, and viscosity enhancers, offering excellent mechanical properties and flexibility to microneedles for easier skin insertion [24].

In this study, GM was incorporated into dissolving microneedles (DMN) to prepare a promising formulation aimed to enhance bioavailability. The two commonly used polymers, PVP K-90 and PVA, known for their favorable physical, chemical, and biocompatible properties in the medical and pharmaceutical fields, were employed as the backbone polymer for the DMN [25]. The micro-molding technique was selected as the manufacturing method due to its suitability for mass production at a relatively low cost [26]. The study utilized pyramid-shaped needles, totaling 100 needles, with the potential for improved penetration and drug release. The MNs (microneedles) were assessed for their mechanical properties, morphology, drug release profiles, and skin penetration. The performance in terms of pharmacokinetic parameters of the developed MNs was compared with that of commercially available glimepiride oral product in healthy human volunteers.

## 2. Materials and Methods

### 2.1. Chemicals and Reagents

Glimepiride (GM, 99% pure) (Lot#: GA2941) was gifted by Oakdale Pharmaceuticals, Hayatabad, Peshawar, Khyber Pakhtunkhwa, Pakistan. Micropoint Technologies Pvt, Ltd. (Singapore) provided Polydimethylsiloxane (PDMS) molds with 1 × 1 cm, 100 cavities of 600 µm in height, 200 µm in base width, and a pitch of 500 µm Soluplus^®^ (Lot#: 85937736WO; BASF; Florham Park, NJ, USA), PVP K-90 (Lot#: SLOBOV; MERCK Sigma-Aldrich; Darmstadt, Germany), PVA (Poly Vinyl Alcohol) (Lot#: P01380a2; MERCK Sigma-Aldrich; Darmstadt, Germany), Acetonitrile (HPLC grade) (Lot#: 1878549; Fisher Scientific; Pittsburgh, PA, USA), Methanol (purity > 99.9%), (Lot#: 1905879; MERCK Sigma-Aldrich; Darmstadt, Germany), Dichloromethane (Lot#: DR0440-001; Fairfield, CA, USA), Sodium Dihydrogen Phosphate (Lot#: K2711145; MERCK Sigma-Aldrich; Darmstadt, Germany), Ortho-phosphoric Acid (Lot#: 1-00573-1000; MERCK Sigma-Aldrich; Darmstadt, Germany), Sodium Bicarbonate (NaHCO3), 99.95% purity (Lot#: SLBR9495V; MERCK Sigma-Aldrich; Darmstadt, Germany), Sodium Chloride (Lot#: S9888; MERCK Sigma-Aldrich; Darmstadt, Germany), Potassium Chloride (Lot#: SZBD1740V, Scharlau Chemie, Barcelona, Spain), Di-sodium Hydrogen Phosphate (Lot#: 1-06586-0500; MERCK Sigma-Aldrich; Darmstadt, Germany), Di-potassium Hydrogen Phosphate (Lot#: 60221, MERCK Sigma-Aldrich; Darmstadt, Germany), Dialysis Tubing-Visking, (MWCO; 12−14 kDa); (Dia = 27/32″–21.5 mm); (Size 6 Inf. 30 M, Sigma-Aldrich; Darmstadt, Germany).

### 2.2. Instruments

Analytical balance (Shimadzu, Kyoto, Japan); bath sonicator (Hwashin Technology Co., Yeongcheon-si, Republic of Korea); vacuum filtration assembly (Sigma-Aldrich, Darmstadt, Germany); distilled water (Millipore ultrapure water system (Milford, CT, USA)). pH meter (Jenway, Essex, UK), magnetic stirrer (DLAB, Fontana, CA, USA), peristaltic pump (Longer Precision Pump Co., Ltd., Baoding, China), centrifuge (DLAB, CA, USA), scanning electron microscope (JSM-5910, JEOL Ltd., Tokyo, Japan), zeta sizer (Malvern Zetasizer ZS-90, Malvern Instruments Ltd., Malvern, UK), FTIR spectrophotometer (Shimadzu, Kyoto, Japan, IRTracer-100), UV spectrophotometer (Perkin Elmer Series 200, Lambda 25, PerkinElmer, Waltham, MA, USA), Franz diffusion cell (Perme Gear, Hellertown, PA, USA), Simultaneous Thermal Analyzer (STA) 8000 by Perkin Elmer (Waltham, MA, USA). The Perkin Elmer Series 200 HPLC system (Norwalk, CT, USA) is coupled with UV–Visible detector, autosampler, in-line degasser, and column oven. The stationary phase used was SUPLECO C18 column (250 × 4.6 mm, 5 μm) and connected via network chromatography interface (NCI 900). Spring applicator (20 mm dia. × 70 mm L; approx. 1.6 N) was purchased from Micropoint Technologies Pte, Ltd. (Singapore).

### 2.3. Methadology

#### 2.3.1. Pre-Formulation Studies

##### Fourier Transform Infrared (FTIR) Analysis

FT-IR analyses were performed to check possible interactions between GM and the added ingredients used in the preparation of microneedles. Two mg GM was mixed with each of the components (PVA, PVP, and Soluplus^®^) at an appropriate ratio, equivalent to that used in the formulation. The spectra of the pure GM, Soluplus^®^, PVA, and PVP K-90 and their physical mixture were analyzed by scanning across a frequency span ranging from 4000–400 cm^−1^ [27].

##### Simultaneous Thermal Analysis, STA (DSC/TGA)

Simultaneous Thermal Analysis techniques, differential scanning calorimetry (DSC), and thermogravimetric analysis (TGA) were utilized to investigate the thermal properties of glimepiride as well as its physical mixture with PVP K-90, PVA, and Soluplus^®^. The STA (Simultaneous Thermal Analyzer-8000) (Perkin Elmer, Waltham, MA, USA) was used for this analysis. DSC and TGA methods were employed to record the thermal transitions and behavior of each sample. These techniques measure the amount of heat needed to raise temperature of the sample and weight loss of the sample from ambient temperature to 900 °C in a dynamic nitrogen atmosphere with a flow rate of 30 mL/min and a heating rate of 10 °C/min.

#### 2.3.2. Solubility Studies of Glimepiride

The effect of different concentrations of Soluplus^®^ (*n* = 3), i.e., 1%, 2%, 4%, 6%, 8%, and 10% *w*/*v*, were tested to achieve maximum solubility and to optimize the Soluplus^®^ concentration as reported earlier [28,29]. An excess amount of drug was gradually added to each 5 mL different concentration of Soluplus^®^ aqueous dispersion to create saturated micelles [30]. The dispersions were covered with Parafilm M and stirred on a magnetic stirrer for 24 h at 25 °C [31]. The mixture was centrifuged at 13,000 rpm for 1 h at 10 °C to separate undissolved drug, and the collected supernatant was filtered through a 0.45 μm filter, diluted with acetonitrile (ACN) and analyzed using HPLC. The optimized micellar solution was used for fabrication of MNs [32]. The size, polydispersity index (PDI), and surface charge (zeta potential) of glimepiride loaded Soluplus^®^ nanomicelles before and after incorporation into dissolving microneedles array was measured using dynamic light scattering via zeta analyzer [33].

#### 2.3.3. Preparation of Microneedle Casting Solution

The drug–Soluplus^®^ aqueous dispersion was prepared by mixing appropriate amount of glimepiride in Soluplus^®^ 10% *w*/*v* in distilled water. Aqueous solutions of PVP-K-90 (40% *w*/*w*) and PVA (11% *w*/*w*) were prepared by dissolving separately each polymer in 5 mL of distilled water, pre-heating to 80 °C, and mixing for six hours to obtain a clear gel-like solution [34]. The polymer solutions were mixed in a 2:1, followed by the addition of a drug–Soluplus^®^ solution while stirring at 150 rpm for 5 min at room temperature using a magnetic stirrer to obtain 1:1 mixture. The drug–polymer blend was left for 24 h to eliminate any air bubbles before fabrication of MNs [34].

#### 2.3.4. Fabrication of GM-Loaded DMN Array

**Figure 2:**
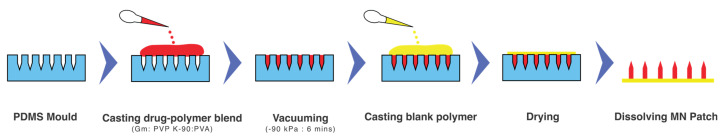
Schematics of the fabrication process of Gm–Nanomicelle-loaded DMNs.

Microneedles were prepared with pure GM without using Soluplus^®^, and GM-loaded Soluplus^®^ nanomicelle microneedles were fabricated using micromolding technique, with a 10 × 10 array of 100 microneedles [35]. The drug–polymer blend (GM: PVP K-90: PVA) in a 1:1 was incorporated into the microarray mold [25,36] applying a vacuum of −90 kPa for about 6 min at room temperature using an oil-less vacuum pump to ensure optimal filling [37]. A drug-free backing layer of PVP K-90 and PVA was then added to the mold and subjected to vacuum under the same conditions [28]. The array was left to dry for 24 h at room temperature [38] and were manually detached from molds with an adhesive tape and stored in a control conditions of temperature and humidity [25]. Figure 2 is an illustration of the fabrication process of Gm–Nanomicelle-loaded DMNs.

#### 2.3.5. Characterization of GM-Loaded MN Array

##### Mechanical Strength Testing

**Figure 3:**
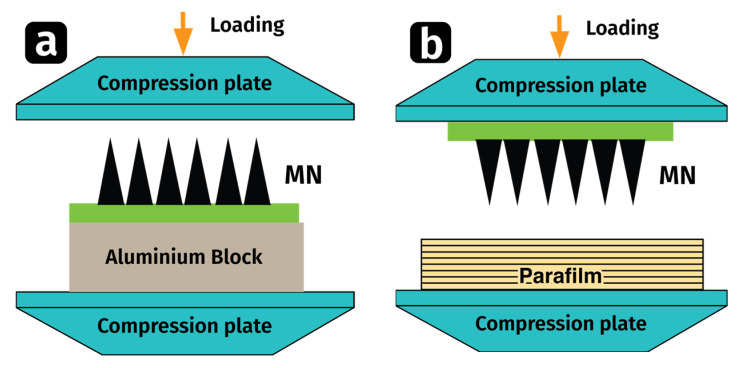
A schematic illustration of Mechanical Strength Testing: (**a**) Fracture Test; (**b**) Insertion Test.

The mechanical properties of the prepared MNs were evaluated (*n* = 3) using the Universal Testing Machine (UTM). Fracture and insertion testing are commonly used methods to evaluate the mechanical strength of microneedles. Fracture testing measures the force required to break or fracture the microneedle, while insertion testing assesses the ability of the microneedle to penetrate the skin without being damaged. Figure 3 showcases two types of Mechanical Strength Testing.

- Fracture Test of GM-Array

The fabricated MNs (*n* = 3) were analyzed for the fracture test using the Universal Testing Machine (UTM) in its compression mode [19]. The MNs were evaluated both with the application of fixed force and free force. In fixed force testing, a predetermined force (32 N) was applied to the MN array. In free force testing, a load was applied gradually on a MN array until the microneedles showed deformation or fractured.

The GM-loaded DMN array was placed on the flat aluminum block, and a stainless-steel cylindrical probe at a rate of 0.5 mm/s for 30 s was pressed against the tips facing upward at a pre-set force of 32 N to record the deflection force [16,39]. The morphology of the MN patch after the test was observed through microscopy. The same was procedure was used in free force testing until the force reached to cause the MNs to fracture.

- Insertion Test of GM-Array

The skin insertion test [40] was performed by applying parafilm M^®^ (Bemis Company Inc., Soignies, Belgium), to assess the insertion properties of DMN arrays. The MN array (*n* = 3) was attached to the stainless-steel cylindrical movable probe of the UTM, and the probe was lowered onto the folded (eight-layered) Parafilm M^®^ at a rate of 0.5 mm/s until a force of 32 N was reached and maintained for 30 s [41]. The MNs were then removed and the number of holes in each layer was counted [42] and % insertion was determined using Equation (1).

(1) $$\mathrm{Percent}\;\mathrm{Insertion}=\frac{\mathrm{Number}\;\mathrm{of}\;\mathrm{perforations}\;\mathrm{created}\;\mathrm{in}\;\mathrm{the}\;\mathrm{parafilm}\;\times \;100}{\mathrm{Total}\;\mathrm{no}.\;\mathrm{of}\;\mathrm{holes}\;\mathrm{microneedles}}$$

##### Scanning Electron Microscopy (SEM)

The morphology and dimensions of microneedles were confirmed using scanning electron microscope. SEM (JSM-5910, Jeol, Japan) was used for the imaging of microneedles at magnifications of up to 300,000×. The fabricated MN patches (*n* = 6) were mounted on the brass stub using graphite glue, coated with gold under vacuum [43], and visualized for images.

#### 2.3.6. Estimation of Drug Content

The glimepiride content in a dissolving microneedle patch (*n* = 3) was determined by soaking the patch in a 10 mL Eppendorf tube filled with distilled water and shaking it in a water bath at 85 °C for 24 h (to enhance dissolution) [44]. The resulting mixture was centrifuged for 20 min at 25 °C and 5000 rpm, followed by reconstitution of the formed precipitate in mobile phase for HPLC analysis [45]. The HPLC system used was a Perkin Elmer Series 200, equipped with a SUPELCO C18 column and a mobile phase consisting of acetonitrile and 8 mM phosphate buffer (Sodium Dihydrogen Phosphate) pH 2.5 (45:55 *v*/*v*) with flow rate of 1 mL/min. A 50 μL volume of the sample was analyzed at 228 nm using a UV detector [46]. The drug content was determined as percent recovery using Equation (2) [47].

(2) $$\mathrm{Percent}\;\mathrm{Recovery}=\frac{\mathrm{Amount}\;\mathrm{of}\;\mathrm{drug}\;\mathrm{recovered}\;\mathrm{on}\;\mathrm{purification}\;\times \;100}{\mathrm{Amount}\;\mathrm{of}\;\mathrm{drug}\;\mathrm{originally}\;\mathrm{taken}}$$

#### 2.3.7. In Vitro Drug Release

**Figure 4:**
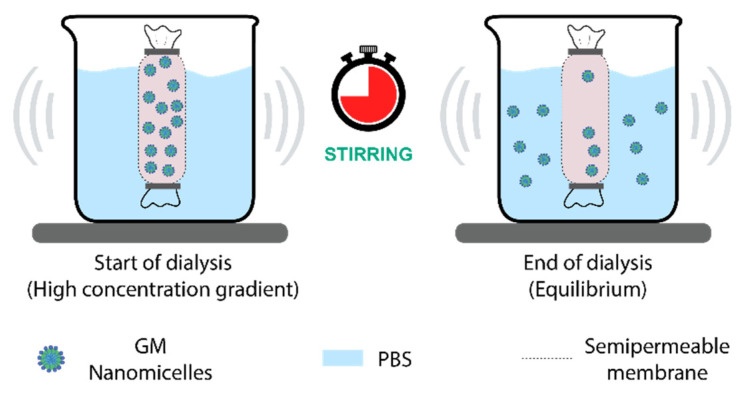
Schematic of in vitro release experimental setup for GM nanomicelles.

The in vitro release from GM nanomicelles and GM–Soluplus^®^-loaded nanomicelles MNS array was investigated. The drug release study on GM nanmicelles was carried out using method described by Pignatello et al. [29]. A 1 mL volume of GM loaded Soluplus^®^ nanomicelles was added to Dialysis Tubing-Visking with a MWCO of 12–14 kDa (27/32″) (21.5 mm), previously soaked in PBS overnight, and dialyzed with PBS pH 7.4 37 ± 1 °C, stirred on magnetic stirrer at 50 rpm. At specific time intervals, i.e., 0.5, 1, 2, 4, 6, 12, and 24 h, a 0.5 mL sample of the release medium was collected and immediately replaced with fresh prewarmed PBS. The samples were suitably diluted and analyzed using HPLC, as outlined in Section 2.3.6. Figure 4 depicts the schematic illustration of in vitro release setup for GM nanomicelles.

**Figure 5:**
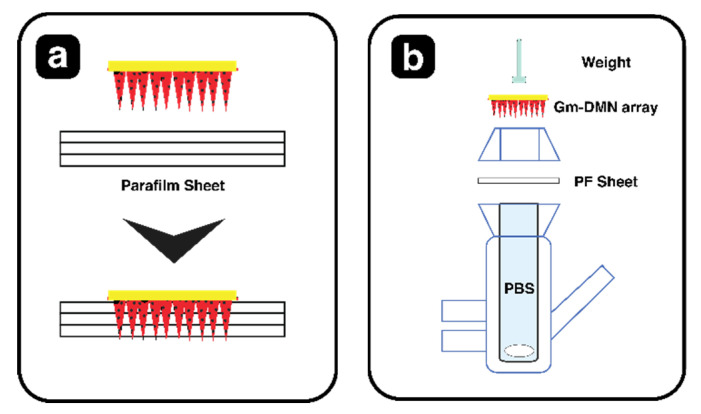
Schematic of the Franz cell experimental setup: (**a**) Insertion of MN array into three prepared stacked layers of PF; (**b**) Disassembled view of vertical Franz diffusion cell.

To evaluate the drug release of GM–DMN (*n* = 3), a Franz diffusion method as shown in Figure 5 was employed.

The three layers of Parafilm M (PF) were stacked, and MN array was inserted via an applicator into the Parafilm M [48]. PF offers a standardized and reproducible substrate, eliminating biological variations. It is cost-effective, easily accessible, and addresses ethical concerns while reducing the risk of infectious diseases. Additionally, it provides greater consistency compared to animal skin [49]. Subsequently, the donor compartment was mounted onto the receptor compartment and filled with phosphate-buffered solution (pH 7.4), stirred at 500 rpm, while keeping the temperature at 37 °C ± 0.5 by circulating warm. Samples (0.5 mL) were collected from receptor compartment at predetermined time intervals, i.e., 0.5, 1, 2, 4, 6, 12, 24, 36, 48, 72, 96, 120, and 144 h, and substituted with an equal volume of dissolution medium [50]. Furthermore, the samples were appropriately diluted (with the mobile phase) and analyzed for drug content at 228 nm using HPLC, following the methodology outlined in Section 2.3.6.

#### 2.3.8. In Vivo Evaluation of Gm–DMNs in Human Volunteers

**Figure 6:**
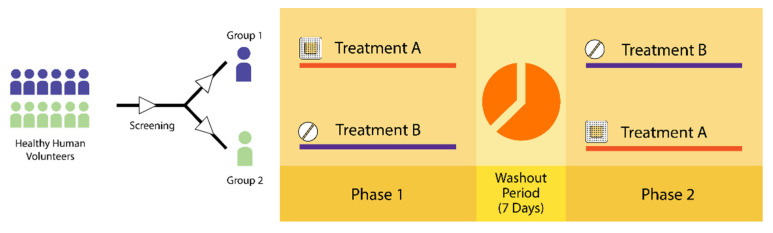
Schematic of the in vivo study design. Two experimental groups were created: Group 1 received MN patch of 240 μg of GM and Group 2 received oral GM of 1 mg (*n* = 6 each) during Phase 1 (and vice versa for Phase 2), having a washout period of 7 days.

Human volunteers were used for the in vivo evaluation to compare the pharmacokinetic parameters of GM-DMNs with the commercially available GM Tablet [51]. Six healthy male volunteers were selected based on medical history and physical examination, with a mean age of 21 ± 0.4 years, a mean height of 174.5 ± 3.8 cm, and a mean weight of 70.6 ± 1.6 kg [52]. The cross-over study was designed with a 7-day washout period to mitigate the influence of potential confounding variables, such as age, body weight, and genetics, that could significantly affect the outcomes of the study [53]. The volunteers were divided into two groups each group comprising of six volunteers. During phase 1, the first group (*n* = 6) received fabricated microneedles containing approximately 240 μg of GM, and the microneedles patches were applied at a force of 1.6 N with a spring applicator (Micropoint Technologies Pvt, Ltd. Singapore), and the second group (*n* = 6) received 1 mg GM oral tablets, as illustrated in Figure 6, and vice versa after the washout time period.

Blood samples (5 cc) from the forearm vein before administration, and at 0.5, 1, 2, 4, 6, 12, 24, 36, and 48 h, postdosing was collected with a 22-gauge needle and transferred into EDTA tubes, plasma was collected from the samples by centrifuging at 1500× *g* for 15 min at 4 °C [52]. The plasma samples were stored at −20 °C prior to analysis. The drug from the plasma was extracted using liquid–liquid extraction by adding 600 μL of methanol to 300 μL of plasma, vortexing for 3 min, and then centrifuging at 10 °C for 15 min at 5500 rpm. The procedure was repeated after the collection of supernatant [54]. The samples were dried under nitrogen at 40 °C and reconstituted with acetonitrile for analysis using HPLC. The study was approved by the ethical committee of Department of Pharmacy, University of Peshawar, approval number 504/EC-FLES-UOP/2022.

#### 2.3.9. In Vivo Safety Assessment

**Figure 7:**
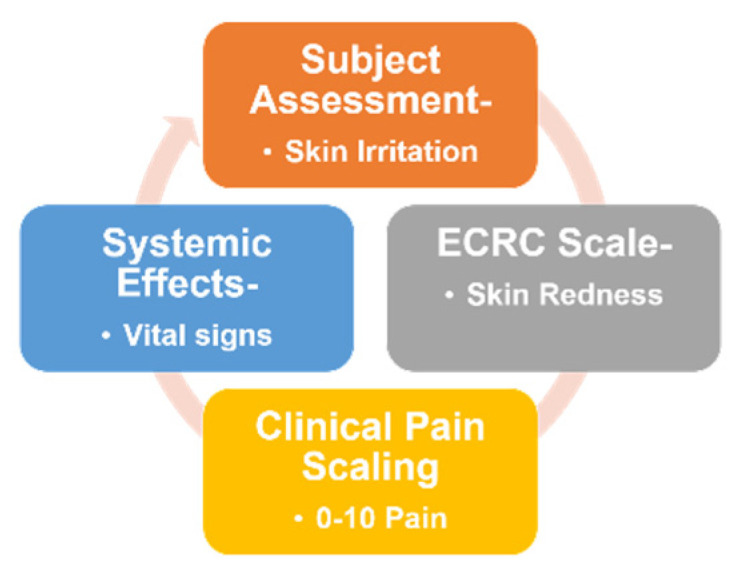
Safety assessment of microneedle arrays.

The safety profile of the MNs was assessed for skin irritation, skin redness (ECRC scale), clinical pain scaling (0–10), and systemic effects (vital signs) as shown in Figure 7 by applying the microneedles to human volunteers (*n* = 6), and the microneedles were left in place for 48 h. Skin inspections were conducted to identify any signs of redness, itching, burning, irritation, or discomfort. Skin redness was evaluated using the ECRC scale. Pain assessment was carried out by asking the participants to rate their pain using a clinical pain scale, ranging from minimal or no pain to unbearable pain requiring immediate medical attention. Systemic effects were assessed by recording vital parameters, i.e., blood pressure, heart rate, and body temperature. These measurements aimed to evaluate any potential impacts on the overall health of the individuals and contribute to the safety profile assessment of the microneedles [55].

### 2.4. Data Analysis

All samples were collected in triplicates, and the data was presented as mean ± standard deviation (SD). Statistical analysis was performed using GraphPad Prism (Version 8.0.2), applying a *t*-test to compare groups. Statistical significance was determined by considering a *p*-value of < 0.05.

## 3. Results

### 3.1. Results and Discussion

#### 3.1.1. Fourier Transform Infrared Analysis (FTIR)

The FTIR spectrum of glimepiride (GM), PVA, PVP K-90, Soluplus^®^, and their physical mixture were obtained using FTIR spectrophotometer (Shimadzu, Kyoto, Japan, IRTracer-100), and upon interpretation, all the characteristic peaks were recorded for GM, PVP K-90, PVA, and Soluplus^®^ as FTIR spectra [8]. It was also noticed that the characteristic peaks were present in the physical mixture as well. The functional groups identified are presented in Table 1.

**Figure 8:**
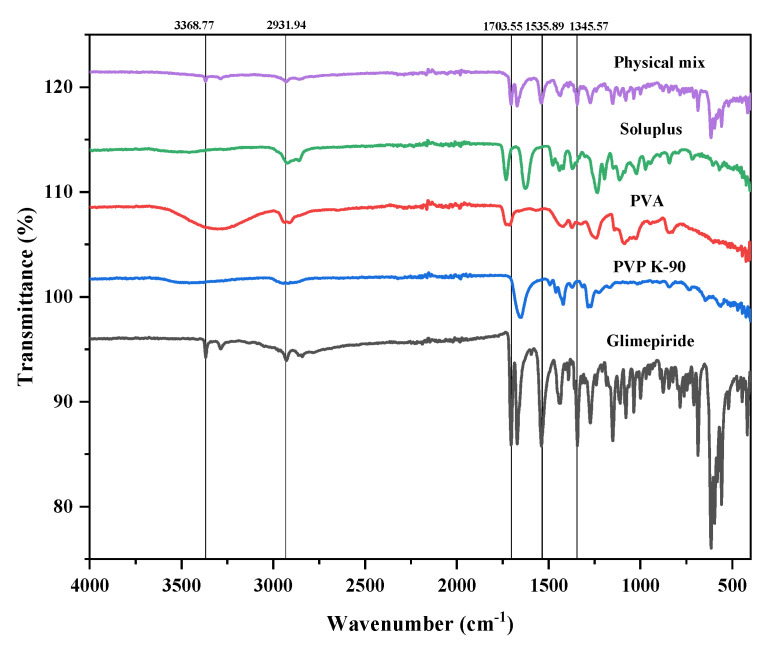
Fourier transform infrared spectra of glimepiride, excipients, and physical mixture.

The FTIR spectral analysis indicated that there were no changes in the characteristic peaks of the pure drug or the polymers in the physical mixture and suggested that there was no observed chemical interaction between drug and the polymers. The characteristic stretching band involving C-H and O-H ranging from 3240–3540 cm^−1^, observed in the PVA spectrum, exhibits reduction in intensity of the physical mixture. This decrease indicates a physical interaction between the drug and polymer, as depicted in Figure 8.

#### 3.1.2. Simultaneous Thermal Analysis, STA (DSC/TGA)

**Figure 9:**
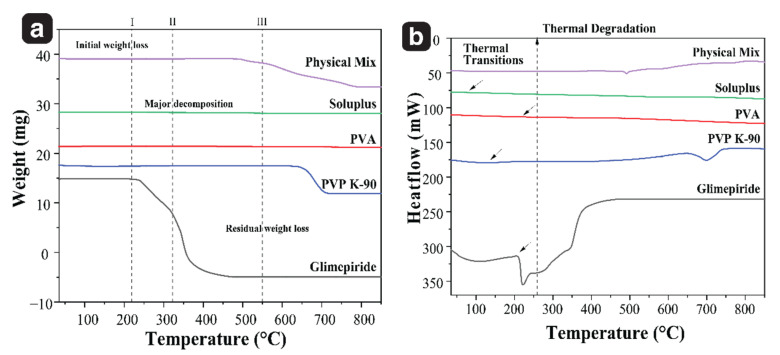
(**a**) Thermogravimetric analysis (TGA) curves of glimepiride, PVP K-90, PVA, Soluplus^®^, and physical mixture. (**b**) Differential scanning calorimetry (DSC) thermograms of glimepiride, PVP K-90, PVA, Soluplus^®^, and physical mixture. Arrows point to the melting points of GM (205 °C), PVP K-90 (135 °C), PVA (209 °C), and Soluplus® (83 °C), respectively.

The DSC and TGA analysis were carried out for the pure drug, excipients, and physical mixtures [8]. TGA of glimepiride indicated that its initial weight loss occurs at 150 °C due to the evaporation of water and other impurities, while major degradation takes place at 300–400 °C due to the decomposition of the glimepiride molecule. The residual weight loss above 500 °C is caused by the oxidation of low molecular weight fragments. When glimepiride is mixed with PVP K-90, PVA, and Soluplus^®^, it showed thermal stability up to 500 °C, indicating that the polymer impart stability to the GM. The physical mixture of drug, PVP K-90, PVA, and SP^®^ retained thermal stability up till 500 °C, as shown in Figure 9a. Table 2, provides the weight loss values at specific temperature.

The DSC curve of pure glimepiride revealed that it undergoes thermal transitions at around 70 °C and has a melting point of 205 °C, beyond which it undergoes thermal degradation at temperatures above 260 °C. This indicated that glimepiride is a crystalline material that melts sharply upon heating. Table 3 presents data on thermal transitions, melting points, and thermal degradations of drug, PVP K-90, PVA, and Soluplus^®^ [27]. The physical mixture of glimepiride and its excipients exhibited a noticeable shift in the melting point, indicating enhanced thermal stability and preventing drug degradation at 205 °C, as evident from the thermogram illustrated in above Figure 9b.

#### 3.1.3. Optimization of GM–Nanomicelles (GNM)

**Figure 10:**
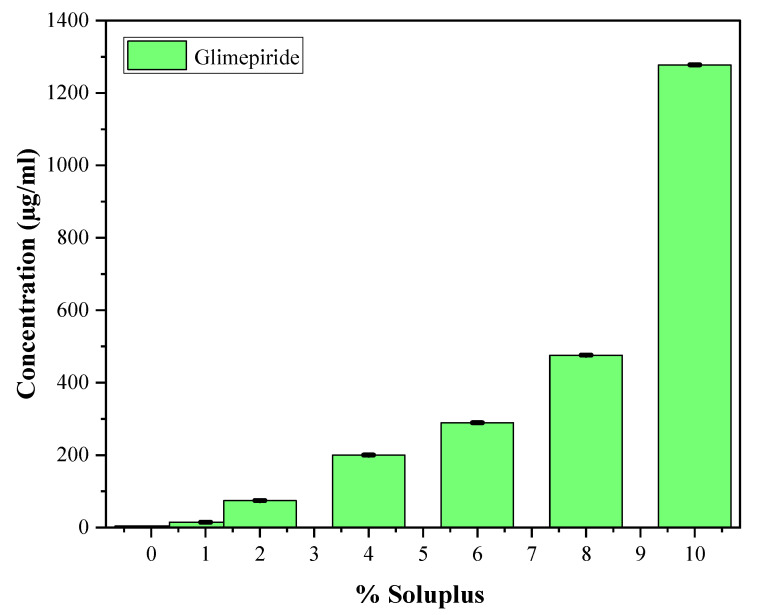
Effect of Soluplus^®^ concentration on the solubility of glimepiride.

The prepared glimepiride nanomicelles (GNM) were incorporated into DMN arrays improving the solubility and bioavailability of the drug. As evident in Figure 10, the highest GNM solubilization (0.4 g/mL) was obtained with 10% (*w*/*v*) Soluplus^®^ concentration [56].

The resultant GNM were of small size (82.6 ± 0.54) and sufficiently high zeta potential (−16.2 ± 0.18) that prevent coalescence of individual particles and result in stable formulation with improved pharmacokinetic properties. The particle size and the zeta potential (ZP) of GNMs shown in Table 4 were measured directly after preparation [57].

**Figure 11:**
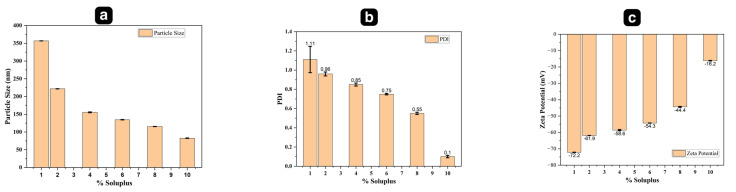
GM–Nanomicelle evaluation: (**a**) Particle size; (**b**) Polydispersity index; (**c**) Zeta potential.

The stability of GNMs was evaluated based on particle size, PDI, and zeta potential as shown in Figure 11a–c.

The highest value of zeta potential (−72 mV) was obtained with 1% Soluplus^®^ concentration and also the particle size was largest (356.8 ± 0.05 nm), which is not desirable, and the PDI value (1.11 ± 0.14) was higher as well, while on the other hand, small particle size (82.6 ± 0.54) was observed with 10% (*w*/*v*) Soluplus^®^ concentration, which is desirable. Though the zeta potential (−16.2 ± 0.18) was low as compared to 1% (*w*/*v*) Soluplus^®^ but still sufficiently high to yield monodisperse nanomicelles as suggested by the PDI value, i.e., 0.1 ± 0.01 [58]. The addition of Soluplus^®^ (SP) to glimepiride at concentrations above the critical micelle concentration (CMC) demonstrated a substantial improvement in aqueous solubility [59]. The solubilization capacity of SP was most pronounced at a concentration of 10% (*w*/*v*). Glimepiride is a neutral molecule without a significant net charge on its surface [60]. However, it can engage in interactions with SP via hydrogen bonding, van der Waals forces, and other non-electrostatic forces. As a result, the overall net charge of glimepiride nanomicelles (GNM) depends on the delicate balance between electrostatic and non-electrostatic interactions between drug and polymer. The negative net charge of nanomicelles suggested that the SP shell possesses a greater negative charge compared to the drug molecules [61,62]. There is polar functional group in the Soluplus^®^ that interact with water molecules to form a hydrated shell, which also contributes to the negative zeta potential [63]. The decrease in negative potential (−72 mV to −16.2 mV) is due to the higher availability of hydrophobic segments, promoting the formation of micelles with more hydrophobic regions. This facilitates the encapsulation of glimepiride molecules shielding the negatively charged groups (OH), resulting in a reduced negative potential of the nanomicelles evident from Figure 11c. However, Figure 11a indicates that with increase in Soluplus^®^ concentration, the particle size decreases subsequently. Table 5 presents the particle size, PDI, and zeta potential of the GM nanomicelles before and after incorporation into DMN array.

Hence, Soluplus^®^ (10% *w*/*v*) was considered optimum concentration for solubilization of GM and incorporation of GM-loaded nanomicelles into the dissolving microneedles array.

#### 3.1.4. Fabrication of Glimepiride Nanomicelle-Loaded DMN Array

**Figure 12:**
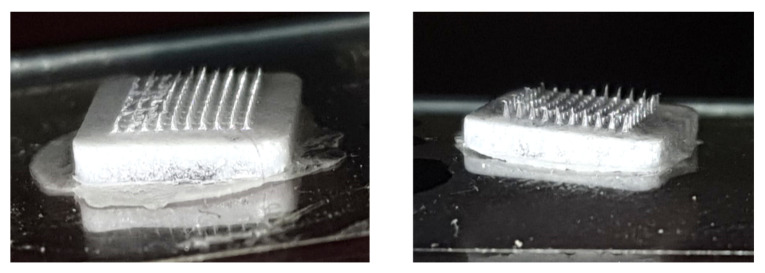
GM microneedles without Soluplus^®^.

Fabrication of microneedles with pure GM without using Soluplus^®^ (SP) was unsuccessful due to their fragility, presence of vacant cavities, and brittleness as evident in Figure 12.

Hence, SP was used to improve the physically stability and uniform distribution, as GM was solubilized. The addition of Soluplus^®^ resulted is mechanically strong and well-formed MNs

**Figure 13:**
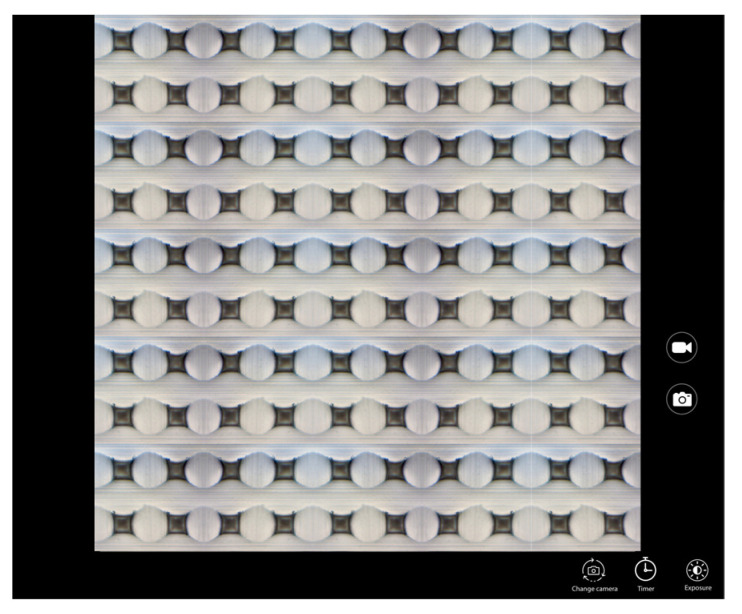
Optical microscope image of a 10 × 10 array.

The microneedles were fabricated using solvent casting method with a PDMS (10 × 10) microarray mold. The polymer solution composed of 2:1 PVP K-90 (40% *w*/*w*) and PVA (11% *w*/*w*) loaded with GM–Soluplus^®^ nanomicelles was filled in the microarray template. The selection of the ratio between PVP K-90 and PVA was determined through a screening process based on considerations of the physical strength and morphology of the microneedles (MNs). These specific concentrations demonstrated optimal outcomes to achieve the desired mechanical strength required for high-quality MNs. The formation of the microneedles were confirmed via microscopy (Figure 13). The morphology and size of the formed DMN was confirmed using SEM.

#### 3.1.5. Scanning Electron Microscopy (SEM)

**Figure 14:**
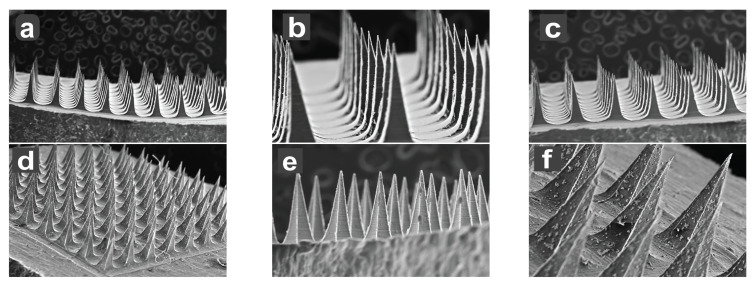
Representative SEM images of GM–DMN patches showing different angle views and magnifications. Ventral views: (**a**) (10×, 500 µm), (**b**) (3×, 100 µm), (**c**) (6×, 500 µm); lateral views: (**d**) (10×, 500 µm), (**e**) (10×, 200 µm), (**f**) (10×, 100 µm) respectively.

The GM–MN were fabricated using 10 × 10 template with 600 µm height, 200 µm base, and 500 µm pitch of pyramid shape. The SEM (Figure 14) studies confirmed the morphology with one evenly distributed pyramid shape microneedle projections. The formed needles had four smooth corners with broad base tapered to the top forming sharp tips. The SEM image depicts the specific dimensions of the MNs in the patch. The formed needles have base with a facet width of 200 µm, which narrows to a fine tip over a length of 600 µm, suggesting that the MNs were of a suitable morphology and height to penetrate the stratum corneum efficiently without causing any discomfort or pain [43].

Figure 14 describes a set of SEM images that display the ventral and lateral views of the GM–DMN array at different magnifications. Image (a–c) show a ventral view of the array at a magnification of 10 × 10, 3 × 10, and 6 × 10 at a scale of 500 µm, 100 µm, and 500 µm, respectively. The images (d–f) represent a lateral view of the array at a magnification of 10 × 10 at scales of 500 µm, 200 µm, and 100 µm, respectively.

#### 3.1.6. Mechanical Strength Testing

The Mechanical Strength Testing provided valuable insight into the mechanical properties of microneedle patches. It is essential for the microneedles to exhibit sufficient mechanical strength to puncture the skin without encountering any mechanical failures and pain.

##### Fracture Test of GM-Array

**Figure 15:**
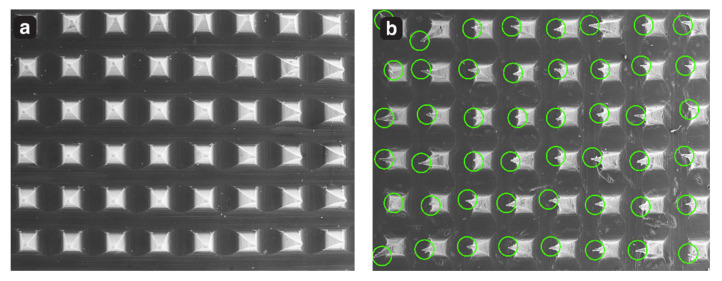
(**a**) Microneedle patch before force application and (**b**) DMN patch after force application with the green circles indicating tips deformation.

The data obtained showed that the prepared microneedle GM–DMN patch withstands a 32 N axial force applied perpendicularly to the patch without any needle fracture. The obtained results were consistent with the previous research conducted [64]. The SEM images are depicted in Figure 15a, b of before and after the test. Figure 15b indicates the deformation of the microneedle tips, but with no cracking or breakage of the needles, suggesting that the DMN had sufficient strength to penetrate the stratum corneum without any needle failure.

The MN patches (*n* = 3) were also subjected to free force up to 285 N (2.85 N/needle) at which the needles deformed without breaking. This indicated that the patch had a high mechanical strength and could withstand a considerable amount of force before reaching its breaking point. The combination of PVP K-90, PVA, and Soluplus^®^ in microneedles synergistically enhances their mechanical strength by leveraging their complementary properties, promoting intermolecular interactions, facilitating better polymer matrix formation, and reinforcing structural integrity [65].

##### Insertion Test of GM-Array

**Figure 16:**
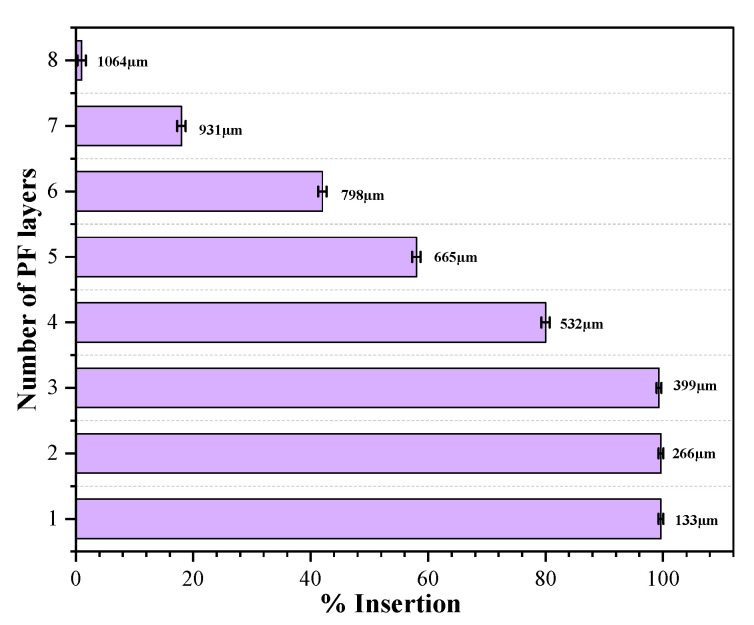
Parafilm M insertion capability of GM–DMN patch.

The number of perforations per PF sheets were counted to determine the insertion efficiency of the fabricated DMN. The PF sheets were checked under the microscope to visualize the perforations. The eight-layered PF had thickness of around 1064 µm with individual layer of 133 µm. It was observed that all the microneedles had penetrated up till third layer of PF, confirming a complete insertion (10%) with depth of 399 μm, while 80% insertion was observed in the fourth layer, as shown in Figure 16.

With a needle height of 600 μm, the MN array can easily pierce the human stratum corneum as the thickness of SC is around 10–20 μm, even the fabricated DMN can easily penetrate deeper into the dermis, which is consistent with the previous study by Zhang et al., 2022 [42].

#### 3.1.7. Estimation of Drug Content

The drug content (*n* = 3) was determined to confirm the uniform distribution of the drug within the DMN. The content was determined using HPLC as discussed in Section 2.3.6 [47] and found to be 100% ± 0.30, i.e., 80.3 µg of GM per array. A drug loading of 80.3 μg was achieved by incorporating a 40% solution of GM-loaded nanomicelles into the total mass (7.6 mg) of the 1 × 1 cm microneedle array. This level of drug loading aligns with findings from previously published literature [16,66].

#### 3.1.8. In Vitro Drug Release

**Figure 17:**
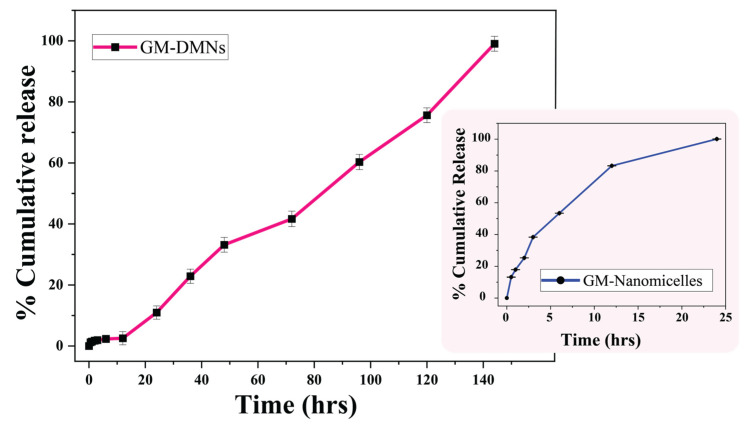
Cumulative drug release profile of GM-dissolving microneedles.

The in vitro release was performed for glimepiride nanomicelles (GNM) and GM-loaded Soluplus^®^ nanomicelles loaded DMNs. The cumulative drug release of glimepiride nanomicelles prior to their incorporation into DMNs and subsequent to their loading is shown in Figure 17.

The GM-loaded nanomicelles were able to sustained the drug release for 24 h, with 83% of the drug is released within 12 h. While the drug was released slowly in a predictable manner for 144 h from nanomicelle-loaded MNs, with 75% of drug released in 120 h.

The drug release kinetics was determined by fitting various mathematical models to the accumulative drug release data. The regression equation and their corresponding R-squared values for the different models are shown in Table 6.

**Figure 18:**
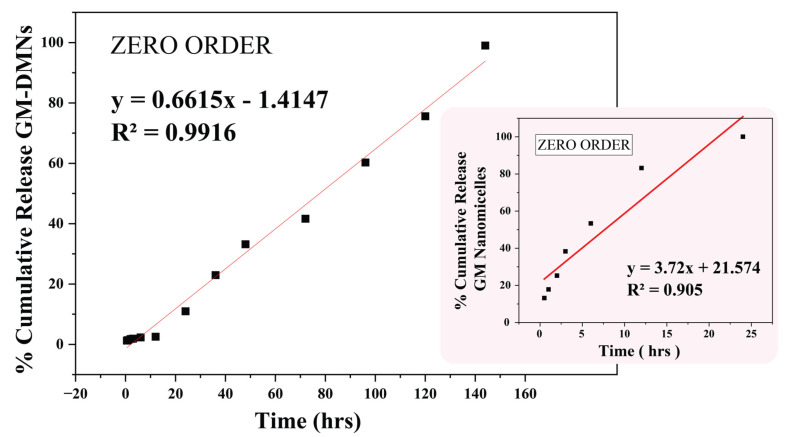
Zero-order kinetics for release from GNM and GM–DMN array.

The zero-order model was found to be the best fit for the drug release in both nanomicelles and DMNs (R^2^ = 0.9916 and 0.905), suggesting that the dissolution of glimepiride from the PVP K-90: PVA patch and GM nanomicelles is concentration-independent, which is the most desirable pattern [67] as depicted in Figure 18.

Hixon–Crowell model was the best fit for the release data with the R^2^ of 0.9871 and 0.8603 for nanomicelles and nanomicelle-loaded MNs, respectively [28]. The results indicated that the GM from the GM nanomicelles and nanomicelle-loaded MNs is released via dissolution of the polymeric matrix; this is in agreement with the drug release from Soluplus^®^ nanomicelles and the PVP K-90 matrix reported earlier [29,68]. The *n* values of 0.536 and 0.883 obtained from the Korsemeyer and Peppas model indicated that the drug release followed a non-Fickian or anomalous transport. The drug release from nanomicelles loaded MNs might be due to the diffusion of a drug molecules and the relaxation of a PVP K-90: PVA matrix undergoing swelling or degradation, leading to the controlled release of the drug [50].

#### 3.1.9. In Vivo PK Evaluation of GM–DMNs in Human Volunteers

**Figure 19:**
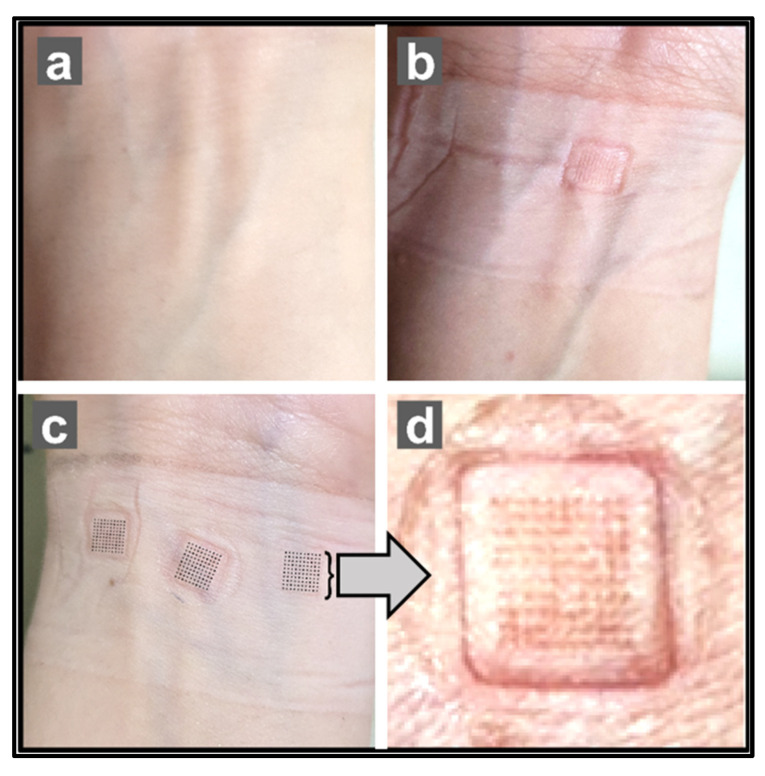
Wrist images illustrating (**a**) pre-patch insertion, (**b**) one patch insertion, (**c**) three GM–DMNs patch application, and (**d**) enlarged GM–DMNs.

The study involved comparison of the pharmacokinetic parameters of the commercially available glimepiride oral formulation (1 mg tablet) and the fabricated dissolving MN patches. The pharmacokinetic parameters were determined for various groups of volunteers after three dissolving MN patches were applied to the wrist (volar part) as shown in Figure 19 via a spring applicator with 1.6 N force and secured with medical tape for 48 h.

**Figure 20:**
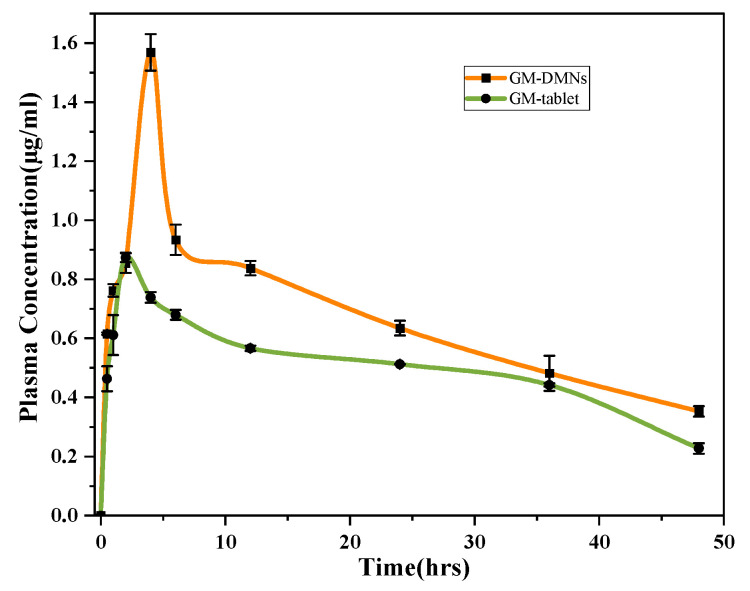
Plasma concentration versus time curve of GM tablet and GM–DMNs.

The pharmacokinetic parameters for the microneedle patch were significantly different (*p* < 0.05) from those of the tablet formulation as shown in Table 7. The C_max_ obtained after administration of DMN was significantly (*p* value = 0.003) higher compared to the oral tablet as depicted in Figure 20.

In contrast to oral administration, the microneedle patch, formulated with nanomicelles, showed a higher C_max_ (1.56 μg/mL ± 0.06) as the solubilized GM in Soluplus^®^ nanomicelles was delivered bypassing the stratum corneum, the major barrier to drug absorption, while in case of tablet, the absorption is restricted as the GM is poorly soluble in the GI fluid and has to pass through the first pass effect, thus resulting in low C_max_ as compared to the DMNs, and the dose of the tablet is high (1 mg) as compared to the low dose (240 µg) of DMN. The plasma samples of the MNs showed the presence of the GM in the plasma over the study period of time with higher AUC and will result in higher bioavailability with lower drug administration as compared to tablets. The higher MRT of DMN is attributed to slow release of drug from the MN patch, resulting in a prolonged stay within the body and a longer half-life. As high concentration (C_max_) of GM was observed with DMN, and GM exhibits >99.5% plasma protein binding, which contributes to its increased T_max_ (4 h) [69].

It is evident that the in vitro drug release of the drug from the MNs is a result of the dissolution of the polymeric matrix. Upon skin insertion, the microneedles absorbed water as both the polymers (PVA & PVPK-90) used are hydrophilic in nature that interact with the skin moisture and adsorb the moisture resulting in the swelling of these polymers. The pyrrolidone group, C_4_H_7_NO, in PVP K-90 readily forms hydrogen bonds with water molecules. These hydrogen bonding interactions between the pyrrolidone groups and water contributed to the swelling behavior of the polymer within the structure of the microneedles. Similarly, when PVA comes into contact with interstitial fluid or skin moisture, hydrogen bonds are formed between the hydroxyl groups of PVA and water, allowing for water absorption and subsequent swelling and dissolution of the polymer subsequently releasing the GM [70]. Simultaneously, microchannels are created, resulting in the ingress of water into the MNs matrix and thus dissolving the polymer and glimepiride to be released. This overall process involves a one-step approach since the microneedle is not removed following application, which can be described as a ‘poke and release’ type mechanism.

The incorporation of Soluplus^®^ as part of the nanomicelles in the microneedle patch played a crucial role in enhancing the penetration of glimepiride through the skin by forming a charged shell and improving solubility and stability [71]. The nanomicelles also resulted in high bioavailability as the portion of DMN that dissolved with in the SC were able to permeate the barrier efficiently. The combination of Soluplus^®^, PVA, and PVP K-90 in the microneedle patch synergistically facilitated drug delivery and modulated drug release while minimizing skin irritation and promoting patient comfort [24].

#### 3.1.10. In Vivo Safety Assessment of Microneedle Arrays in Human Skin

Skin irritation, including itching, burning sensation, and discomfort, was evaluated as “yes” or “no.” Skin redness was scored using the ECRC scale, score 0 representing no redness, while pain experienced by the subjects was assessed on a clinical pain scale. Systemic effects were measured through vital signs, including systolic and diastolic blood pressure (mm of Hg), pulse rate (bpm), and body temperature (°F). The assessment of the subjects’ responses is summarized in Table 8.

The data indicates that none of the subjects reported itching, burning sensation, or discomfort. Skin redness scoring was 0 for all subjects, suggesting no significant skin irritation. Pain scaling was scored as 1 for all participants, indicating minimal or no pain. Vital signs, including systolic and diastolic blood pressure, pulse rate, and body temperature, were within normal ranges for all subjects. These findings contribute to the evaluation of the safety profile of the microneedles and suggest no notable adverse effects on skin or systemic health in the studied population.

## 4. Conclusions

The present study introduces an innovative drug delivery approach by incorporating glimepiride-loaded nanomicelles into dissolving microneedles (DMNs). The nanomicelles are uniformly distributed within the PVP K-90 and PVA polymer matrix, yielding mechanically robust and smooth microneedles, as confirmed by SEM images. The optimized nanomicelles exhibit a favorable particle size (82.6 ± 0.54), PDI (0.1 ± 0.01), and zeta potential (−16.2 ± 0.18), displaying a significant 250-fold increase in solubility compared to previous data. FTIR analysis confirms excipient compatibility with glimepiride, and thermal analysis ensures microneedle stability. In vitro evaluations demonstrated that the release of GM from nanomicelles and nanomicelles-loaded DMN is governed by a concentration-independent, non-Fickian transport mechanism. The microneedles deliver the drug predictably through dissolution of the polymer–drug matrix, as validated by the Hixon–Crowell model. In vivo assessments demonstrated sustained transdermal drug delivery of GM–nanomicelles. The microneedles’ drug release occurs via the dissolution of the polymeric matrix, using a one-step ‘poke and release’ mechanism. Microneedles effectively penetrate the skin barrier, leading to active glimepiride absorption in plasma and displaying improved pharmacokinetic parameters compared to a 1 mg GM oral tablet in healthy human volunteers. This study’s success is using nanomicelles in dissolving microneedles that provides a more efficient and controlled drug release, with the potential to enhance transdermal delivery and drug absorption, offering benefits for glycemic control in diabetic patients with low dose and dosing frequency. High drug loading surpasses previous levels, setting a new standard for drug-loaded microneedles, promising improved therapeutic efficacy and dosage accuracy for various medical conditions, including diabetes treatment. The safety assessment indicated the microneedles’ safety with no adverse effects on the skin or health.

## Figures and Tables

**Table 1:** Glimepiride and excipients FTIR spectra interpretation.

Glimepiride	PVP K-90	PVA	Soluplus^®^	Physical Mixture	Interpretation
3368.77	3364.42	-	3368.86	3368.86	N-H stretch (secondary amine)
2931.94	2934.38	2932.25	2932.18	2932.18	C-H stretch (aliphatic)
1704.93	1707.50	1705.25	1705.05	1705.05	C=O stretch
1670.95	1656.91	-	-	1672.87	N-C=O stretch

**Table 2:** TGA outcomes: I: initial weight loss, II: major decomposition, and III: residual weight loss.

	I: Initial Weight Loss	II: Major Decomposition	III: Residual Weight Loss
Glimepiride	Approx. 150 °C	324 °C	Above 500 °C
PVP K-90	Approx. 50 °C	350 °C	Above 600 °C
PVA	Approx. 65 °C	270 °C	Above 700 °C
Soluplus®	Approx. 50 °C	330 °C	Above 570 °C

**Table 3:** DSC outcomes: thermal transitions, melting points, and thermal degradation.

	Thermal Transitions	Melting Points	Thermal Degradation
Glimepiride	Approx. 70 °C	205 °C	Above 260 °C
PVP K-90	Approx. 100 °C	135 °C	Above 250 °C
PVA	Approx. 60 °C	209 °C	Above 220 °C
Soluplus^®^	Approx. 50 °C	83 °C	Above 200 °C

**Table 4:** Influence of different conc. Of SP on particle size, PDI, and zeta potential of GM–nanomicelles.

SP% (*w*/*v*)	Particle Size (d·nm)	PDI	Zeta Potential (mV)
1	356.8 ± 0.05	1.11 ± 0.14	−72.2 ± 0.14
2	221.8 ± 0.21	0.96 ± 0.02	−61.9 ± 0.07
4	155.5 ± 1.04	0.85 ± 0.01	−58.6 ± 0.38
6	134.6 ± 0.51	0.75 ± 0.01	−54.3 ± 0.10
8	115.5 ± 0.21	0.55 ± 0.01	−44.4 ± 0.31
10	82.6 ± 0.54	0.1 ± 0.01	−16.2 ± 0.18

**Table 5:** Particle size, PDI, and zeta potential before and after incorporation into DMN Array.

Parameters	GM–Nanomicelles	GM–DMNs
Particle size (d·nm)	82.6 ± 0.54	83.1 ± 0.14
PDI	0.1 ± 0.01	0.1 ± 0.10
Zeta (mV)	−16.2 ± 0.18	−16.8 ± 0.70

**Table 6:** R-square values, K values, N values, and regression equations obtained by fitting kinetic models.

	GM–Nanomicelles				GM–DMNs Array			
Model	R^2^	K	N	Regression Equation	R^2^	K	N	Regression Equation
Zero order	0.905	3.72	--	y = 3.72x + 21.574	0.9916	0.66	--	y = 0.6615x − 1.4147
First order	0.435	0.06	--	y = 0.067x + 0.768	0.8316	0.01	--	y = 0.014x + 0.3519
Higuchi	0.9055	−1.11	--	y = −1.114x + 5.725	0.7158	−0.22	--	y = −0.2229x + 5.1692
Hixson and Crowell	0.9871	−0.20	--	y = −0.205x + 4.650	0.8603	−0.01	--	y = −0.0196x + 4.8235
Korsemeyer and Peppas	0.8091	--	0.536	y = 1.313x + 0.536	0.9298	--	0.883	y = 0.8829x − 0.0589

**Table 7:** PK parameters for GM tablet compared to fabricated GM–DMN patch.

Parameters	Units	Oral Tablet 1 mg	GM–DMN Patch 240 µg	*p*-Value
		Mean ± SD	Mean ± SD	
t_1/2_	h	29.19 ± 1.96	27.33 ± 3.17	0.33
T_max_	h	2 ± 0	4 ± 0	0 *
C_max_	μg/mL	0.87 ± 0.01	1.56 ± 0.06	0.003 *
AUC 0-t	μg/mL·h	24.11 ± 0.04	32.07 ± 0.95	0.009 *
AUMC 0-inf	μg/mL·h^2^	1365.04 ± 166.38	1858.29 ± 271.85	0.055 *
MRT 0-inf	H	40.26 ± 3.05	40.04 ± 3.37	0.905
Vz/F	(μg)/(μg/mL)	1246.15 ± 28.62	204.50 ± 11.24	0.0002 *
Cl/F	(μg)/(μg/mL)/h	29.70 ± 1.25	5.23 ± 0.33	0.0010 *

* *p*-value < 0.05 is significant.

**Table 8:** Safety profile assessment of dissolving microneedles patch.

	Subject Assessment-Skin Irritation			ECRC Scale-Skin Redness	Clinical Pain Scale	Systemic Effects-Vital Signs			
Subjects (Male)	Itching (Yes/No)	Burning Sensation (Yes/No)	Discomfort (Yes/No)	Skin Redness Scoring	Pain Scale Score	Systolic BP (mm of Hg)	Diastolic BP (mm of Hg)	Pulse Rate (bpm)	Body Temp.
**1**	Yes	No	No	0	1	115 ± 5.2	77 ± 2.1	73 ± 1.7	98.9 °F ± 0.6
**2**	No	No	No	0	1	120 ± 7.2	79 ± 4.3	78 ± 3.4	97.9 °F ± 1.8
**3**	Yes	Yes	No	0	1	120 ± 2.8	79 ± 4.8	87 ± 1.4	97.8 °F ± 1.1
**4**	No	No	No	0	1	114 ± 2.4	74 ± 3.1	77 ± 2.2	97.9 °F ± 1.2
**5**	No	No	No	0	1	120 ± 4.1	79 ± 1.6	79 ± 2.7	97.9 °F ± 0.8
**6**	No	No	No	0	1	113 ± 2.9	79 ± 1.4	73 ± 1.4	97.8 °F ± 0.7

## Data Availability

The data are included in the manuscript.
